# Raman Spectroscopy and Machine Learning in the Diagnosis of Breast Cancer

**DOI:** 10.1007/s10103-025-04597-3

**Published:** 2025-09-02

**Authors:** Sowndarya Rao, Nikita Sharma, Vyasraj G Bhat, Vibha Kamath, Mehak Thakur, Sindhoora Kaniyala Melanthota, Subir Das, Budheswar Dehury, Nirmal Mazumder

**Affiliations:** 1 Department of Bioinformatics, Manipal School of Life Sciences, Manipal Academy of Higher Education, Manipal, Karnataka 576104 India; 2 Department of Biophysics, Manipal School of Life Sciences, Manipal Academy of Higher Education, Manipal, Karnataka 576104 India; 3 Department of Chemistry, University of Zurich, Zurich, Switzerland

**Keywords:** Systematic review, Raman spectroscopy, Machine learning, Breast cancer

## Abstract

**Abstract:**

The most prevalent cancer in women worldwide, breast cancer, greatly benefits from early identification for better prognoses. But traditional diagnostic techniques, like biopsies and mammograms, can require invasive procedures and lack accuracy. The non-invasive, quick, and accurate nature of machine learning (ML) and Raman spectroscopy (RS) in breast cancer diagnoses are examined in this review. Combining machine learning’s capacity to analyse intricate spectrum datasets with Raman spectroscopy’s ability to produce molecular fingerprints of biochemical alterations linked to cancer improves diagnostic precision. Using the PRISMA methodology, studies published from 2017 to 2024 were examined, with an emphasis on those that reported sensitivity and specificity values greater than 80%. With sensitivity and specificity frequently over 90%, the nine included studies show that Raman spectroscopy combined with machine learning methods such as support vector machines, convolutional neural networks, and linear discriminant analysis yields good diagnostic metrics. The investigation highlights Raman spectroscopy’s adaptability in analysing biological material, such as tissues and serum, with prospective uses extending to intraoperative, real-time evaluations. Although encouraging, there are still issues that need to be resolved, like the requirement for common frameworks, multi-centre validation, and affordable technology. A thorough assessment of RS-ML applications is given by this study, which also offers insights into its therapeutic potential and directs future studies in breast cancer detection.

**Clinical trial number:**

Not applicable

**Supplementary Information:**

The online version contains supplementary material available at 10.1007/s10103-025-04597-3.

## Introduction

With around 2.3 million new cases diagnosed each year, breast cancer is the most frequent cancer among women and a major cause of cancer-related mortality, thereby making it one of the biggest worldwide health concerns [1]. The likelihood of surviving for people with breast cancer is primarily determined by the stage of diagnosis, even with advancements in treatment methods. Early detection is essential for better survival rates. Advanced-stage disease and mortality may increase as a result of delayed diagnosis and treatment [2]. The use of conventional diagnostic techniques including MRI, ultrasound, and mammography has proved to be crucial for early detection. To validate diagnoses, these techniques rely on invasive procedures like biopsies, but they frequently lack sensitivity and specificity, especially in women who are younger with dense breast tissues [3]. Conventional diagnostic techniques usually lack precision, which could postpone an accurate diagnosis. The gold standard for identifying a range of tumours is still conventional pathology examination, but clinical practice requires a precise, rapid, and non-invasive diagnostic tool [4].

Every biological sample possesses a distinct chemical makeup, resulting in unique vibrational patterns when examined through Raman spectroscopy. These spectral signatures reveal comprehensive molecular details about proteins, carbohydrates, lipids, and nucleic acids within tissues and cells. By identifying these biochemical differences, Raman spectroscopy can accurately distinguish between cancerous and healthy cells and tissues [5]. Because it is non-invasive, requires a little sample preparation, and can analyse a wide range of biological samples, including tissues, serum, and saliva, Raman spectroscopy is a versatile technique for diagnosing breast cancer [6]. The diagnostic capabilities of Raman spectroscopy have been greatly improved by the incorporation of machine learning methods. Large, complicated spectrum datasets can be handled well with machine learning, which makes it possible to recognize patterns and classify them with high sensitivity and specificity [7]. Based on unique molecular signals, Raman spectroscopy has proven to be able to differentiate between different cancerous and non-cancerous cells [8].

Notwithstanding these positive developments, a number of challenges remain until Raman spectroscopy’s clinical use for cancer diagnosis becomes widely accepted. The need for consistent analytical frameworks, differences in spectrum collection techniques, and sample preparation heterogeneity are some of the main obstacles. Furthermore, to validate the diagnostic accuracy and repeatability of Raman spectroscopy across a variety of patient populations, comprehensive, multicentre research is required [6]. The goal of this review is to thoroughly examine research on the use of Raman spectroscopy in conjunction with machine learning approaches for breast cancer diagnosis that was between 2017 and 2024. We want to assess the diagnostic accuracy of Raman spectroscopy, investigate its possible clinical uses, and pinpoint knowledge gaps by combining data from various investigations. By conducting this analysis, we want to offer insights that will direct future investigations and make it easier to include Raman spectroscopy and machine learning into standard clinical procedures for the diagnosis of breast cancer.

### Raman spectroscopy

Named after C.V. Raman, Raman spectroscopy is an analytical method that uses light scattering to quantify the vibrational modes of molecules [9]. The dispersed light, which is a feature of molecular bonding, gives information about the structure of the sample This procedure makes it possible to identify rotational, vibrational and other molecule states, which makes it an effective way to examine chemical compositions [10].

**Fig. 1:**
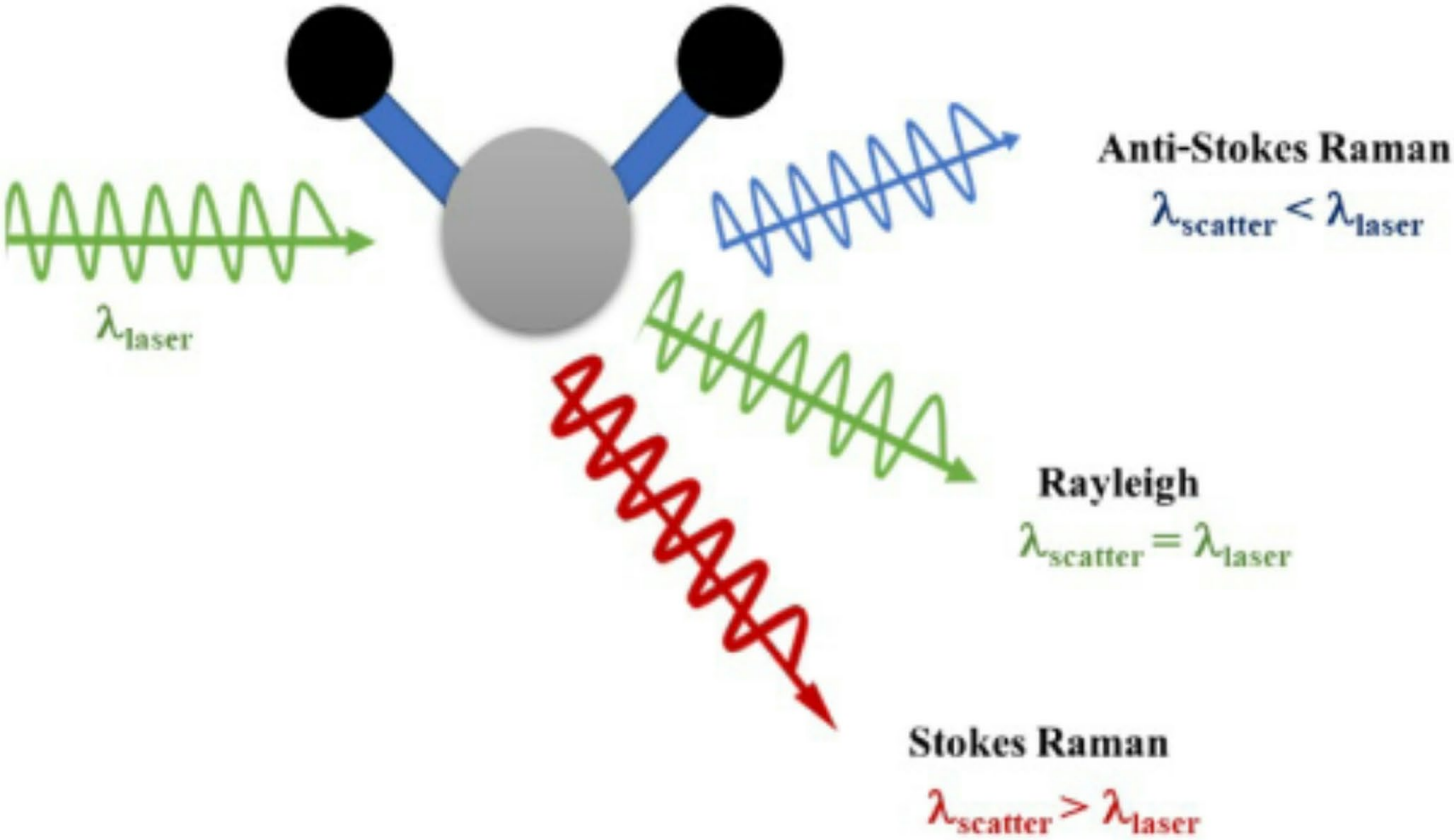
Illustration of Raman scattering showing how molecules interact with excitation light to produce Rayleigh, Stokes, and Anti-Stokes scattering’s effects [11]

There are two types of Raman scattering: inelastic and elastic. There is no change in the light’s wavelength in elastic scattering, like Rayleigh scattering. Depending on the molecular vibrational levels, the scattered light in inelastic scattering, sometimes referred to as Raman scattering, shows a wavelength change. Anti-Stokes Raman scattering (blueshift) occurs when the scattered photon gains energy, while Stokes Raman scattering (redshift) occurs when the photon loses energy (Fig. 1). The difference between incident and scattered light wavelengths is known as the Raman shift, and it is a crucial parameter in Raman spectroscopy. It makes it possible to compare spectra from various lasers in a meaningful way [12].

### Types of Raman spectroscopy

**Fig. 2:**
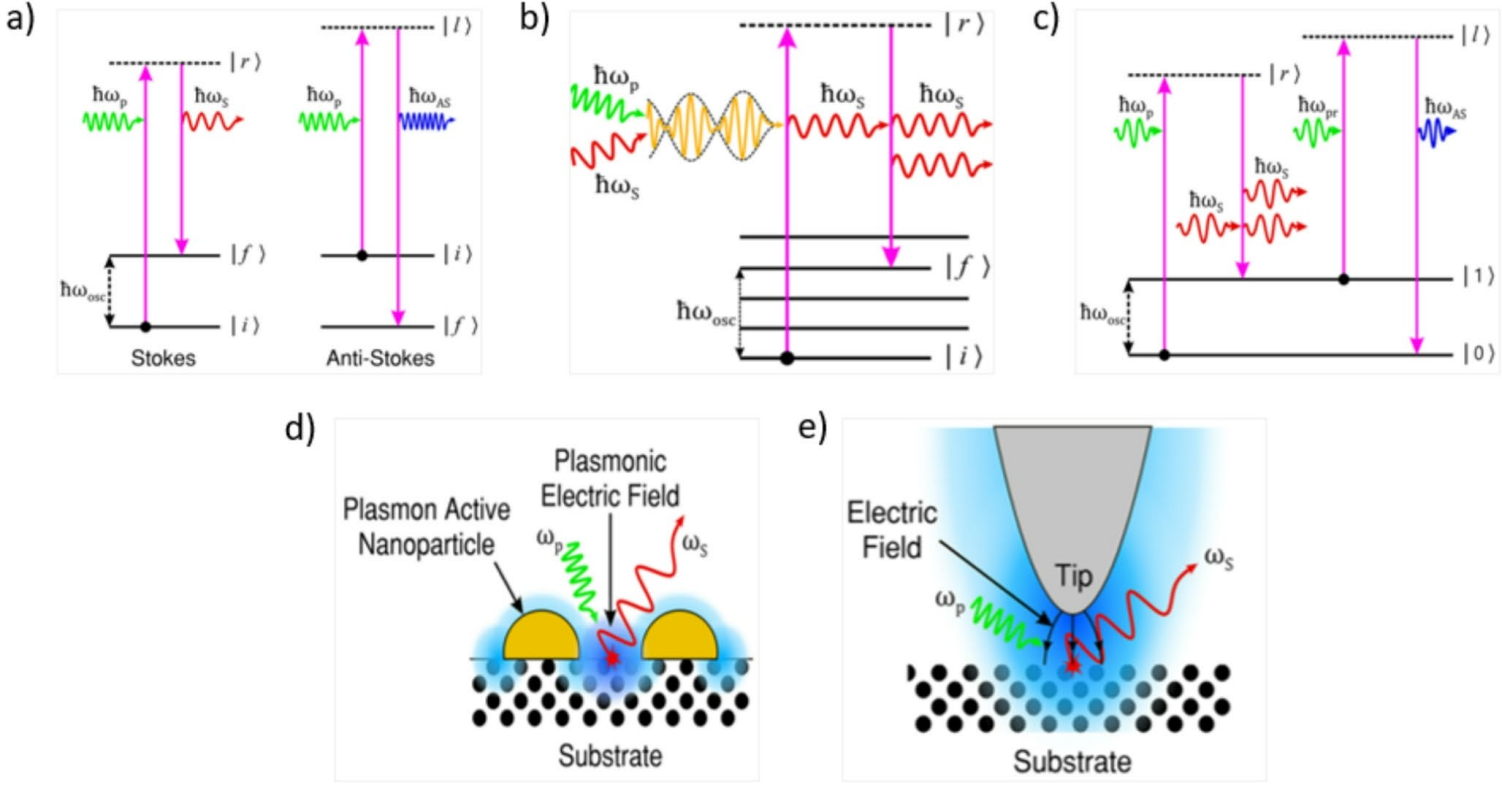
Illustration and explanation of Raman spectroscopy techniques: **a** Spontaneous Raman scattering: shows the process of incoherent scattering where incident photons interact with molecules to produce Stokes and Anti-Stokes transitions driven by molecular vibrations. **b** Stimulated Raman scattering (SRS): Demonstrates coherent Raman scattering where two photons produce a greater vibrational signal, enhancing the detection of molecular interactions. **c** Coherent Anti-Stokes Raman Scattering (CARS): generates a coherent Anti-Stokes signal using multiple photons, allowing for very specific molecular vibrational characterization. **d** Surface-Enhanced Raman Scattering (SERS): improves weak Raman signals using plasmonic nanoparticles on rough metallic surfaces. **e** Tip-Enhanced Raman scattering (TERS): Thid technique offers high-resolution spectral analysis by concentrating localized electromagnetic fields at a pointed tip close to the sample surface [15]

More than 25 specialized methods have been developed using Raman spectroscopy. The incoherent process of spontaneous Raman Scattering causes scattered photons to show changes in energy levels based on molecular interactions. Surface-Enhanced Raman Spectroscopy (SERS) amplifies Raman signals by utilizing nanostructured metallic surfaces, enabling highly sensitive detection of low-concentration biomarkers. Nevertheless, inconsistencies in nanoparticle synthesis pose reproducibility challenges, limiting its reliability [13]. Tip-Enhanced Raman Spectroscopy (TERS) combines scanning probe microscopy with Raman spectroscopy, achieving nanoscale spatial resolution ideal for subcellular imaging. Although it offers high sensitivity, its complex instrumentation and lengthy scanning times hinder its practicality in clinical applications [14]. Coherent Anti-Stokes Raman Scattering (CARS) is a nonlinear Raman method that facilitates label-free, rapid imaging of biological tissues with excellent molecular specificity. However, despite, its fast data acquisition, CARS is affected by background noise, which can cause spectral distortions [15]. Because of its excellent specificity and sensitivity, surface-enhanced Raman spectroscopy (SERS) is one of the most commonly employed method in the detection of early-stage cancer [16]. Figure 2 illustrates various Raman spectroscopy techniques.

### Raman data analysis

Data collection and storage, pre-processing of spectra to account for background noise and normalization, followed by statistical analysis using chemometric techniques, form the essential workflow in Raman data analysis. The workflow typically involves several steps: first, acquiring spectral data from biological samples; second, performing pre-processing procedures such as baseline correction, smoothing, normalization, and removal of cosmic rays; third, extracting relevant spectral features; and finally, applying statistical and machine learning models for classification and interpretation. These steps ensure that spectral data is interpreted accurately and consistently [17, 18].

### Machine learning

**Fig. 3:**
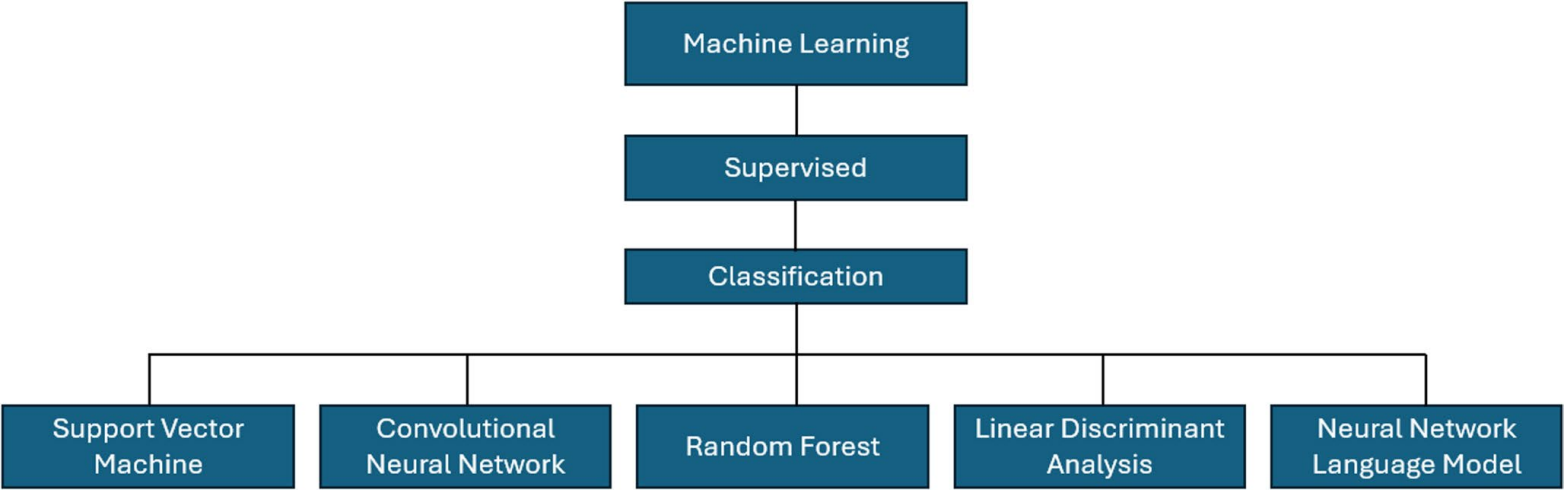
Supervised machine learning classification models applied for breast cancer diagnosis using raman spectroscopy

Machine learning is increasingly used in Raman spectrum analysis, particularly for cancer detection. It involves training algorithms to identify patterns and make predictions from spectral data, enhancing the diagnostic accuracy and efficiency of Raman spectroscopy. Machine learning techniques are broadly categorized into supervised learning, which relies on labeled data for classification and regression tasks, and unsupervised learning, which is used to discover hidden structures in unlabelled data [19]. All the included studies employed supervised learning techniques. The following models were most frequently applied, demonstrating robust diagnostic performance in classifying Raman spectra for breast cancer diagnosis (Fig. 3). Support Vector Machine (SVM) is one of the commonly used models. It finds the best boundary that separates different classes, making it effective for distinguishing between two groups, like cancerous and non-cancerous cells. SVM performs well even with small data sets and is good at avoiding overfitting [7]. Convolutional Neural Networks (CNNs) are another powerful tool, particularly for pattern recognition. They have multiple layers that automatically learn complex features from spectral data, helping to identify intricate patterns and improving classification accuracy [20]. Linear Discriminant Analysis (LDA) is often used to reduce the number of variables by projecting high-dimensional data onto a lower-dimensional space while keeping different classes separate. This makes the classification process more efficient [21]. Random Forest is an ensemble learning method that builds multiple decision trees and uses the majority vote or average prediction for the final result. It is highly accurate, reduces overfitting, and works well with imbalanced datasets, making it suitable for complex spectral data [22]. Neural Network Language Model (NNLM) is designed for sequence prediction tasks and is effective in learning spectral patterns. It predicts subsequent data points by understanding the contextual relationship within spectral sequences [23].

### Meta analysis and PRISMA chart

A statistical method for combining data from several research to produce solid results is called meta-analysis. A systematic review that finds pertinent papers using precise inclusion and exclusion criteria is frequently the first step in the process. The results are then combined, and effect sizes are assessed using statistical methods. To summarize the data and direct therapeutic practice, meta-analyses are crucial in healthcare research. The PRISMA statement provides guidelines for conducting and reporting systematic reviews and meta-analyses [24]. It consists of a flow diagram and an item checklist. It places a strong emphasis on precision, openness, and following established procedures to guarantee that the results are trustworthy and repeatable. PRISMA is frequently used in medical research and is especially helpful for assessing interventional trials [25].

## Method and methodology

### Data collection and search strategy

Using the terms “Raman spectroscopy”, “Machine learning”, and “Breast cancer”, a search was done across four databases – PubMed, Web of Science, Scopus, and Embase in November 2024. All the studies that were found by the search were exported in CSV format. To facilitate processing, these datasets were merged into a single excel file. The review process was organized using the PRISMA methodology. Ethical approval was not necessary because of the nature of the study. The literature search yielded a total of 228 articles. Records identified individually from these different databases are PubMed = 47, Embase = 53, WoS = 68 and Scopus = 60.

### Deduplication

Microsoft Edexcel was used to remove duplicate entries that came from overlapping records. The dataset in the combined excel file was sorted alphabetically, and Conditional Formatting → Highlight Cell Rules → Duplicate Values was applied to automatically flag overlapping records based on title, and author names. These flagged duplicates were then manually verified and removed to ensure accuracy. Out of 228 articles, 68 unique articles were shortlisted after removing redundancy. The full list of all 68 identified studies are included as a supplementary file (Supplementary Table S1).

### Study selection process

Following deduplication, the studies underwent multiple rounds of screening to assess eligibility. Initial filtering was done based on the relevance of the study titles and the Abstract. Finally, a thorough full-text assessment was done to determine the relevant papers.

#### Eligibility criteria

A systematic approach was used to select high-quality studies for the meta-analysis. The selection process involved screening titles and abstracts, reviewing full texts, and strictly following predefined inclusion and exclusion criteria. Studies were included in primary research articles published in peer-reviewed journals between 2017 and 2024, focusing on the use of Raman spectroscopy combined with machine learning for breast cancer detection. Variations of Raman spectroscopy, including SERS, TERS, and CARS, were also considered. Additionally, studies had to report key diagnostic performance metrics, such as sensitivity and specificity. Only those with sensitivity and specificity of at least 80% were included to ensure clinically relevant accuracy. Human serum, tissues, and cell line samples were included in the study. To maintain statistical rigor, studies with sample size (*n* > 30) were included. Studies were excluded if they were review articles, meta-analyses, case reports, or conference abstracts. Non-English publications were not considered. To maintain statistical validity, studies with fewer than 20 cases were excluded. The quality and reproducibility of each selected study were carefully evaluated. Studies lacking full-text availability were excluded to ensure the inclusion of only reliable and reproducible studies.

**Fig. 4:**
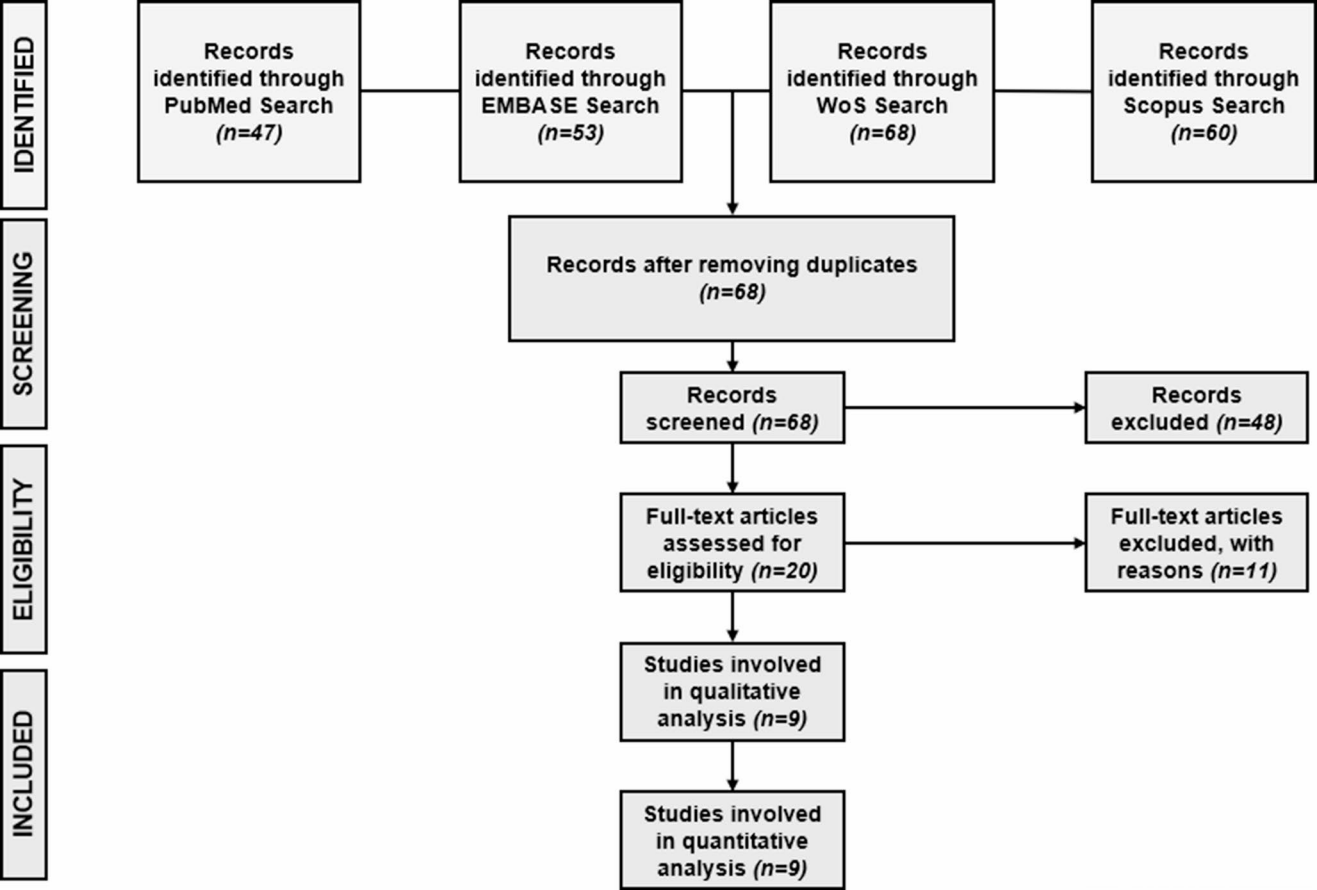
Flow of information through the different stages of a systematic review (Prisma chart)

After screening 68 articles by title and abstract based on the inclusion and exclusion criteria, 20 were selected for further review, and 9 were ultimately included in the meta-analysis. Figure 4 illustrates the PRISMA flowchart, which outlines the systematic review process from initial identification to final selection.

### Statistical analysis

To assess the possibility of publication bias across the included studies, funnel plots were generated. Sensitivity, specificity, and sample size values were extracted for each study, and min-max normalization was applied to ensure comparability. Sensitivity reflects Raman spectroscopy’s ability to correctly identify breast cancer cases (true positives), while specificity indicates its accuracy in identifying non-cancerous cases (true negatives). High sensitivity reduces false negatives, which is critical for early diagnosis, whereas high specificity minimizes false positives, improving overall diagnostic precision. Funnel plots were constructed using the ggplot2 package in R to visualize the relationship between effect size (sensitivity/specificity) on the x-axis and standard error on the y-axis. Additionally, forest plots were created to summarize the diagnostic performance of Raman spectroscopy across studies. These plots present the individual sensitivity and specificity estimates from each study, along with their corresponding 95% confidence intervals (CIs), and include the pooled estimates derived from meta-analysis. In cases where CIs were not reported, they were calculated using the available sensitivity, specificity, and sample size data. A narrow CI indicates greater precision and consistency, while a wider CI suggests higher variability across studies. Forest plots enhance visual interpretation by allowing comparisons of effect sizes and precision across studies.

## Result and discussion

The methods, effectiveness, and therapeutic implications of the combination of Raman spectroscopy and machine learning for breast cancer diagnosis are examined in this review, which extracts its data from nine investigations. With a varied number of sample sizes, the investigations used a variety of sample types, such as serum, breast tissue specimens, blood and cell cultures. Serum’s sensitivity to biochemical changes in cancer patients and simplicity of collection has made it the most useful medium. One of the included studies investigated the application of serum Raman spectroscopy combined with Support Vector Machine (SVM) for rapid breast cancer screening. A total of 333 serum samples were analysed, achieving a sensitivity of 93.2% and specificity of 95.1%. These metrics demonstrated the robustness of SVM in classifying spectral patterns associated with cancer biomarkers, highlighting its potential as a non-invasive diagnostic tool for early cancer detection [26]. In another investigation, serum SERS technology using thermally annealed silver nanoparticle composite substrates was integrated with SVM to detect breast cancer. Despite, a relatively small sample size of 28 serum samples, this approach achieved perfect sensitivity and specificity (100%). The enhanced signal clarity provided by the SERS substrates was attributed to the amplification of cancer-related spectral features, demonstrating the high diagnostic accuracy achievable with optimized sample preparation techniques [27]. To profile tumour heterogeneity and classify breast cancer subtypes, Random Forest was employed with SERS data. Analysing 124 serum samples, the model achieved a sensitivity of 97.8% and specificity of 92.2%. Random Forest’s robustness to overfitting and ability to handle complex spectral variations were evident in its accurate classification of cancer subtypes, underlining its potential for personalized cancer treatment approaches [22].

Another study utilized Convolutional Neural Network (CNN) to diagnose ductal carcinoma in situ and breast cancer from serum samples. This model was tested on a large dataset of 804 serum samples and achieved a sensitivity of 98.5% and specificity of 97.2%, demonstrating high scalability and reliability for population-wide screening. The CNN’s layered architecture enabled effective pattern recognition, ensuring high diagnostic precision [20]. The combination of SERS with a two-dimensional CNN and Gramian Angular Field transformation was evaluated for breast cancer screening using 128 serum samples. This model demonstrated a sensitivity of 98.65% and specificity of 97.67%. The 2D CNN’s capability to capture complex spatial features significantly enhanced classification accuracy, highlighting the advantages of deep learning in spectral image analysis [28]. Finally, transformer based language models was applied for rapid breast cancer diagnosis using 271 serum samples, achieving perfect sensitivity and specificity (100%). The model’s sequence prediction capability effectively captured spectral patterns, showcasing its potential for real-time cancer diagnostics and emphasizing the growing relevance of neural networks in spectral data analysis [23].

Breast tissue samples were primarily used to differentiate malignant tissues from normal tissues while cultured breast cancer and normal mammary epithelial cells were studied to identify biochemical differences. The classification of breast cancer tissue samples was explored using a one-dimensional convolutional neural network (1D-CNN). This model was tested on 40 tissue samples and achieved a sensitivity of 98% and specificity of 86%. The high sensitivity reflected the model’s capability to accurately detect cancerous tissues, though the lower specificity suggested some misclassification of non-cancerous samples. This highlights the importance of further optimization to reduce false-positive rates in tissue diagnostics [29]. Similarly, another study involving tissue samples combined Raman spectroscopy with Support Vector Machine and reported 93% and 95% sensitivity and specificity respectively [30].

Raman micro-spectroscopy combined with Linear Discriminant Analysis (LDA) was utilized to distinguish breast cancer cells from normal mammary epithelial cells. After analyzing 60 cell line samples, this model achieved a sensitivity of 96% and specificity of 100%. The high classification accuracy illustrated LDA’s strength in dimensionality reduction and enhanced class separability, particularly effective in handling high-dimensional spectral data [21].

Many investigations used surface-enhanced Raman spectroscopy (SERS), which improves signal clarity by using substrates based on silver or gold nanoparticles. The spectrum sensitivity and reproducibility were further enhanced by sophisticated substrates designs, such as AgNPs/PSB [27]. Across the reviewed studies, a range of preprocessing methods were applied to enhance Raman spectral data prior to machine learning analysis. For baseline correction, common techniques included asymmetric least squares (ALS) and polynomial fitting [23, 26]. Noise reduction methods included Savitzky-Golay smoothing [23]. For normalization max-min scaling, and area normalization [20] were employed. To extract important features from the spectral data, more sophisticated approaches like GAF (Gramian Angular Field) transformation, dimensionality reduction strategies like PCA and multivariate curve resolution (MCR-ALS) were also used.

While many studies report high diagnostic performance of surface-enhanced Raman spectroscopy (SERS), it is important to interpret such findings with caution. Some works, including [16], report high sensitivity and specificity without addressing potential variability, overfitting, or lack of validation. Recent evaluations [31] have highlighted concerns regarding exaggerated diagnostic claims in SERS-based liquid biopsy studies. These findings underline the need for rigorous standardization, reproducible protocols, and large-scale, blinded validation cohorts to truly establish the clinical utility of SERS in early cancer detection.

The wide range of machine learning models included Convolutional Neural Networks (CNNs) as well as more conventional models like support vector machine (SVM) and PCA-Linear Discriminant Analysis (PCA-LDA). With excellent metrics, the CNN models achieved high sensitivity, specificity and accuracy. In classification accuracy, SVM and 1D-CNN showcased strong performance with augmented datasets, but generally trailed 2D-CNN, which demonstrated robust performance with datasets that are not augmented [28]. The potential of these methods for accurate cancer detection was demonstrated by the fact that accuracy varied from 92 to 100% across trials, with sensitivity and specificity scores often exceeding 90%.

While SVM and CNN models have demonstrated high classification performance in Raman-based breast cancer diagnosis, it is important to acknowledge their limitations. Recent studies have shown that ML models, especially SVM and Random Forest, can produce inflated accuracy metrics when applied to small or non-representative datasets without robust validation (L. A. Bratchenko & Bratchenko [31, 32]. This underscores the necessity of using independent test sets, rigorous cross-validation, and transparency in reporting model training processes to ensure reproducibility and generalizability of results.

**Fig. 5:**
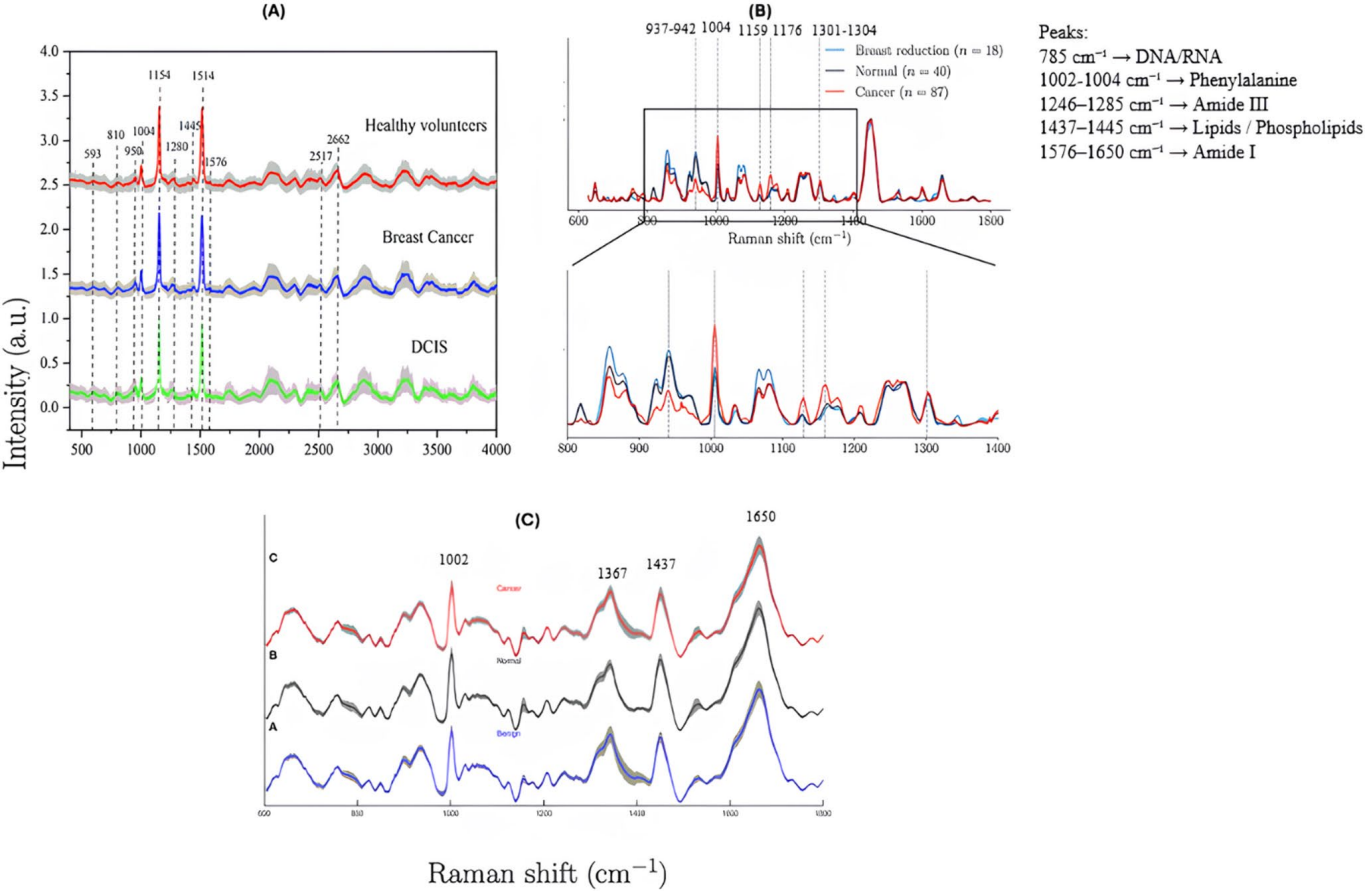
Raman spectroscopy profiles reveal variations in breast tissue conditions across healthy, cancerous, and benign samples. **A** Broad spectral analysis identifies key differences in biomolecular content, with notable peaks at different points in healthy, breast cancer, and DCIS samples. **B** A focused spectral range further highlights differences among breast reduction, normal, and cancer samples, reflecting molecular composition changes. **C** Comparisons of cancerous, normal, and benign tissues show biochemical alterations, with Raman bands linked to proteins, lipids, and nucleic acids acting as potential diagnostic markers [20, 26, 33]

Additionally, a review of the studies included revealed consistent trends in the experimental design and analytical targets. The most frequently employed Raman excitation wavelength was 785 nm, due to its optimal balance between penetration depth, signal intensity, and minimized fluorescence background [20, 28]. Serum was the most commonly analyzed biological matrix, offering a non-invasive, easily obtainable, and biochemically informative medium for detecting cancer-associated metabolic alterations [23, 26]. Important Raman spectral features crucial for differentiating malignant from healthy samples included peaks associated with phenylalanine (~ 1002–1004 cm⁻¹), amide III bands (~ 1246–1285 cm⁻¹), amide I bands (~ 1576–1600 cm⁻¹), lipid and phospholipid-related vibrations (~ 1437–1445 cm⁻¹), and DNA/RNA backbone signals (~ 784–785 cm⁻¹), highlighting their vital roles in cancer detection. (Fig. 5).

The studies revealed an array of advantages, including non-invasiveness, excellent precision in diagnosis, cost effectiveness and adaptability to settings with limited resources. These approaches are a promising addition to conventional diagnostic procedures such as imaging and histology because of their high sensitivity and specificity. Translation into clinical practice is hampered by constraints like small sample size, single-centre datasets, reliance on pre-processing, augmentation, and the requirement for specialized technology. Furthermore, even while experimental results are encouraging, large-scale, multi-centre trials are necessary to validate these approaches and guarantee their generalizability and dependability in clinical settings. Widespread use is also hampered by the requirement for specific substrates and Raman spectrometers, especially in environments with limited resources. Additionally, the review’s inclusion criteria prioritized studies reporting sensitivity and specificity values above 80%, which, while intended to focus on high-performance diagnostic models, may have excluded potentially valuable studies with lower diagnostic metrics or smaller datasets. Future reviews could benefit from incorporating a broader range of studies, including those with modest diagnostic performance, to provide a more comprehensive assessment of the field’s progression.

Despite, all the drawbacks, this analysis highlights how Raman spectroscopy and machine learning have the potential to revolutionize breast cancer diagnostics by providing a non-invasive, effective, and scalable solution that may enhance early detection and supplement conventional techniques.

The details of each individual study have been listed and tabulated in Table 1.

**Table 1:** Summary of studies included in the analysis evaluating Raman spectroscopy for breast cancer detection and diagnosis

Sl. No.	Authors and Publication year	Type of RS used for detection/diagnosis	Laser Wavelength	Power	Sample type	Sensitivity	Specificity	Number of spectra	Machine learning/statistical model used	Total sample size (*n*)	Feature extraction/dimensionality reductionality methods
1.	Lin et al., [26]	Conventional Raman Spectroscopy	785 nm laser	70mW	Human Serum	93.2%	95.1%	3330	Support Vector Machine	333	Prinicipal Component Analysis (PCA)
2.	Cheng et al., [27]	SERS	785 nm laser	160mW	Human serum	100%	100%	165	Support Vector Machine	28	PCA
3.	Ma et al., [29]	Conventional Raman spectroscopy	785 nm laser	Not mentioned	Human tissue	98%	86%	600	One-dimensional Convolutional Neural Network (1D-CNN)	20	1D-CNN
4.	Iwasaki et al., [21]	Raman micro spectroscopy	632.8 nm laser	Not mentioned	Human cell lines	96%	100%	60	Linear Discriminant Analysis	60	MCR-ALS
5.	David et al., [30]	Conventional Raman spectroscopy	785 nm laser	100mW	Human tissue	93%	95%	238	Support Vector Machine	20	SVM
6.	Ishwar et al., [22]	SERS	785 nm laser	5mW	Human serum	97.8%	92.2%	18,600	Random Forest	124	PCA
7.	Wang et al., [6]	Conventional Raman spectroscopy	532 nm laser	8mW	Human serum	98.5%	97.2%	804	Convolutional Neural Network	804	1D-CNN
8.	Cheng et al., [28]	SERS	785 nm laser	Not mentioned	Human serum	98.65%	97.67%	640	Two-dimensional Convolutional neural network	128	GAF- 2D-CNN
9.	Li et al., [23]	Conventional Raman spectroscopy	532 nm laser	20mW	Human serum	100%	100%	813	NNLM	271	PCA

**Fig. 6:**
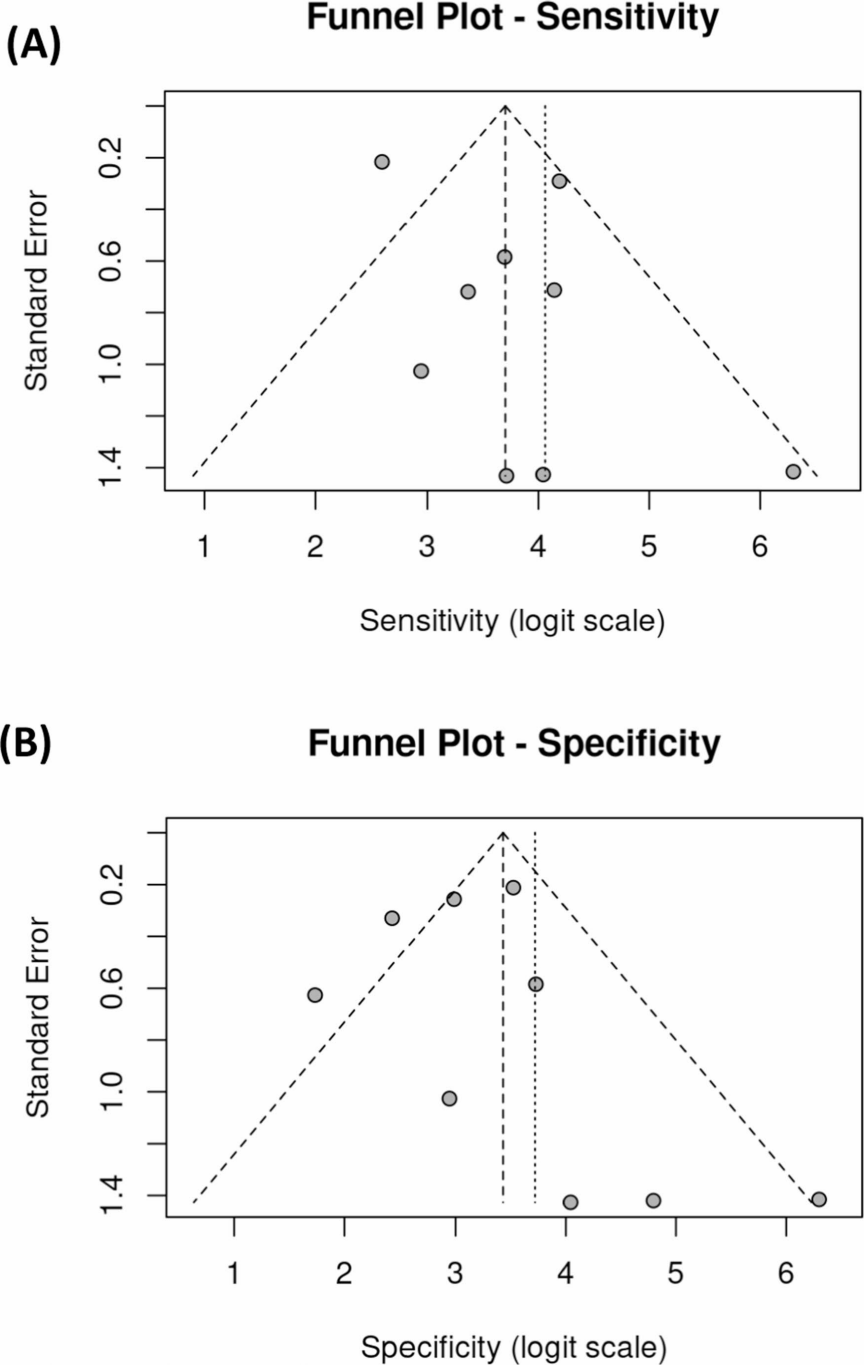
Funnel plots showing the sensitivity and specificity of Raman spectroscopy across nine studies included in the analysis. The x-axes represent sensitivity and specificity values, while the y-axes indicate the standard error of each study. The red dashed lines represent the pooled sensitivity and pooled specificity values, respectively. Figure 6**A** shows a symmetric distribution of studies around the pooled sensitivity estimate, indicating minimal publication bias and high consistency in sensitivity across studies. In contrast, Fig. 6**B** displays slight asymmetry in the specificity plot, suggesting potential mild publication bias or heterogeneity. The observed variability in specificity may be attributed to differences in sample types, experimental protocols, or analytical techniques used in the studies

Potential publication bias in sensitivity and specificity estimates among the included papers was evaluated using funnel plots (Fig. 6A and B). The x-axes show the sensitivity and specificity values, while the y-axes represent the standard error. Panel (A) shows the funnel plot for sensitivity, and Panel (B) presents the funnel plot for specificity. Each black dot corresponds to an individual study, positioned according to its effect size (either sensitivity or specificity) and standard error. The gray triangular area represents the expected distribution of studies if no publication bias is present. The red dashed lines indicate the pooled sensitivity and specificity values. The nearly symmetrical pattern in the sensitivity funnel plot suggests minimal publication bias, as most studies are grouped closely around the pooled estimate. However, the specificity funnel plot shows some asymmetry, suggesting a slight publication bias or variability between studies. This variation could be due to differences in methodologies, sample types, or statistical approaches used in the studies.

**Fig. 7:**
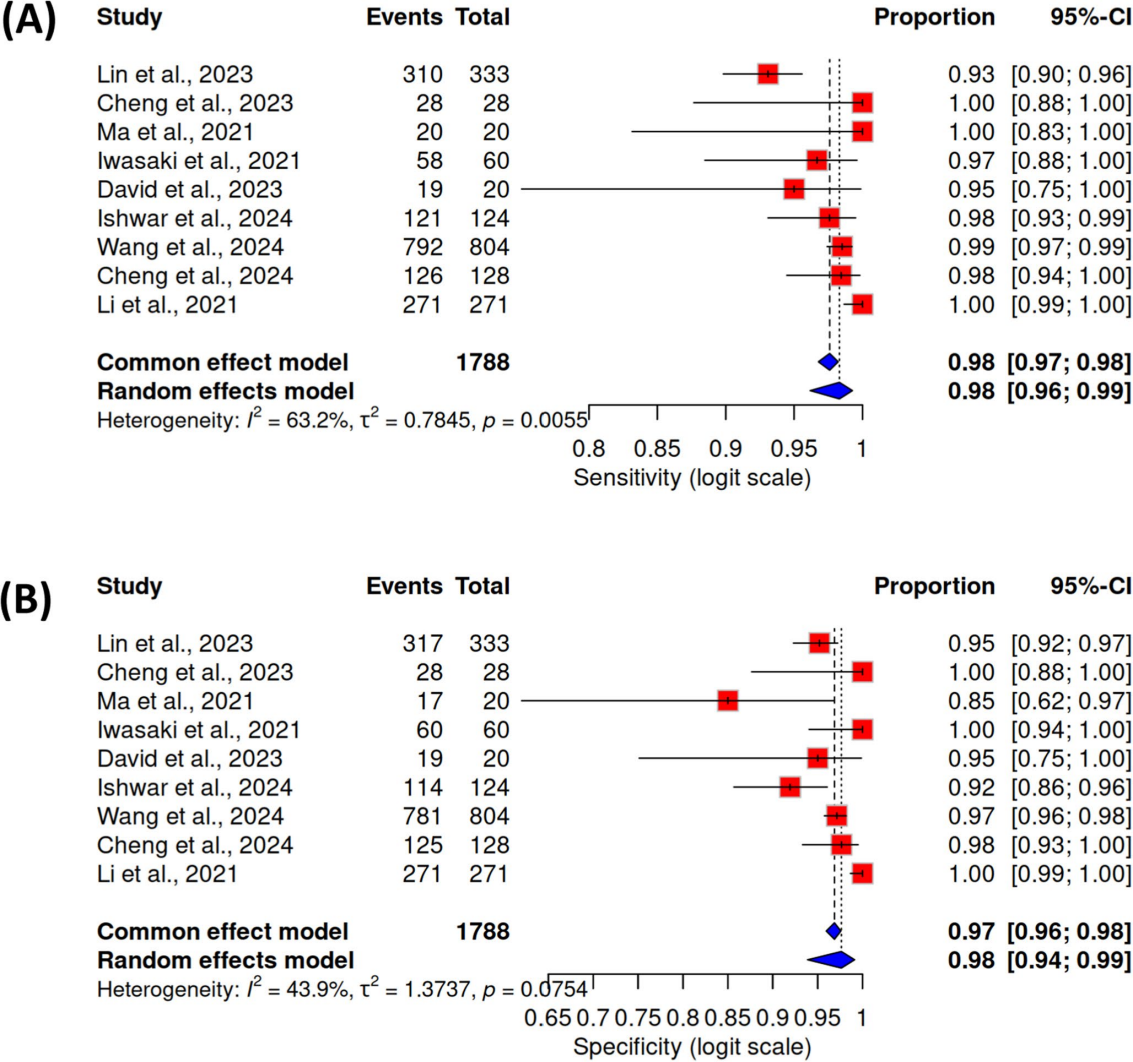
Forest plots showing the sensitivity 7**A** and specificity 7**B** of Raman spectroscopy-based breast cancer diagnosis across nine studies. Each horizontal line represents a study’s 95% confidence interval, with the central marker indicating its point estimate. The diamond at the bottom of each plot represents the pooled mean estimate with its corresponding 95% confidence interval, calculated using a random-effects meta-analysis. Slight extension of some confidence intervals beyond 1.0 reflects estimation from high-performing models and small sample sizes, handled using continuity correction

In addition to funnel plot analysis, forest plots were generated to visualize the pooled diagnostic performance of Raman spectroscopy across the included studies (Fig. 7A and B). Using a random-effects meta-analysis model, individual and pooled sensitivity and specificity estimates were displayed alongside their 95% confidence intervals. The pooled sensitivity was 0.98 (95% CI: 0.97–1.00), and the pooled specificity was 0.97 (95% CI: 0.96–0.99), confirming strong diagnostic consistency across studies.

## Conclusion

The combination of Raman spectroscopy and machine learning offers a non-invasive, accurate, and scalable approach to diagnosing breast cancer. Across the included studies, supervised learning models—such as SVM, Random Forest, CNNs, and LDA—achieved high diagnostic performance, with sensitivity and specificity frequently exceeding 90% [20, 26, 28]. The most frequently used excitation wavelength was 785 nm, chosen for its optimal balance between signal strength and minimal fluorescence interference [20, 28]. Serum emerged as the most effective and scalable sample type due to its non-invasive nature and biochemical richness [23, 26]. Key Raman biomarkers—such as phenylalanine (~ 1004 cm⁻¹), collagen and lipids (~ 1445 cm⁻¹), tryptophan (~ 1365 cm⁻¹), and protein-related amide bands (~ 1217–1683 cm⁻¹)—were consistently associated with malignant changes [20, 33, 34]. While tissue-based approaches offer high accuracy, their invasive nature limits their applicability. Non-invasive approaches using serum and saliva show great promise for large-scale screening. However, several challenges remain, including a lack of standardization in spectral acquisition, preprocessing protocols, and limited multicentre validation. Addressing these issues through uniform data acquisition protocols and large-scale trials will be essential for clinical translation. In conclusion, Raman spectroscopy presents a groundbreaking approach to breast cancer diagnosis by enhancing early detection and improving treatment outcomes.

## Supplementary Information

Below is the link to the electronic supplementary material.

ESM 1 (DOCX 36.8 KB)

## Data Availability

The data can be available upon request to corresponding author.
