# Supplementary material for: Raman Spectroscopy and Machine Learning in the Diagnosis of Breast Cancer
Source: Lasers Med Sci. 2025 Sep 2;40(1):348. doi: 10.1007/s10103-025-04597-3 (PMC12405035; doi:10.1007/s10103-025-04597-3)
Supplement: Supplementary file 1 — (DOCX 36.8 KB) [file 10103_2025_4597_MOESM1_ESM.docx]

| **Raman spectroscopy and Machine learning in the diagnosis of Breast cancer**  **Table S1: List of all 68 studies identified during the systematic literature search** |
| --- |

| **Title** | **Year** |
| --- | --- |
| 3D Superclusters with Hybrid Bioinks for Early Detection in Breast Cancer | 2024 |
| A Molecular Typing Method for Invasive Breast Cancer by Serum Raman Spectroscopy | 2024 |
| Accurate diagnosis of lung tissues for 2D Raman spectrogram by deep learning based on short-time Fourier transform | 2021 |
| Activation of epidermal growth factor receptors in triple-negative breast cancer cells by morphine; analysis through Raman spectroscopy and machine learning | 2024 |
| Advancing cancer diagnostics with artificial intelligence and spectroscopy: identifying chemical changes associated with breast cancer | 2019 |
| Application of serum Raman spectroscopy combined with classification model for rapid breast cancer screening | 2023 |
| Application of serum SERS technology based on thermally annealed silver nanoparticle composite substrate in breast cancer | 2023 |
| Cancer Stem Cell Derived Extracellular Vesicles with Self-Functionalized 3D Nanosensor for Real-Time Cancer Diagnosis: Eliminating the Roadblocks in Liquid Biopsy | 2022 |
| Cervical cancer diagnosis model using spontaneous Raman and Coherent anti-Stokes Raman spectroscopy with artificial intelligence | 2025 |
| Classifying breast cancer tissue by Raman spectroscopy with one-dimensional convolutional neural network | 2021 |
| Classifying Raman spectra of extracellular vesicles based on convolutional neural networks for prostate cancer detection | 2020 |
| Comment on “Serum Raman spectroscopy combined with multiple classification models for rapid diagnosis of breast cancer” | 2023 |
| Deep convolutional neural networks as a unified solution for Raman spectroscopy-based classification in biomedical applications | 2022 |
| Deep learning-assisted monitoring of trastuzumab efficacy in HER2-Overexpressing breast cancer via SERS immunoassays of tumor-derived urinary exosomal biomarkers | 2024 |
| Detection of acquired radioresistance in breast cancer cell lines using Raman spectroscopy and machine learning | 2021 |
| Diagnosis of pregnancy disorder in the first-trimester patient plasma with Raman spectroscopy and protein analysis | 2024 |
| Dimensional reduction based on peak fitting of Raman micro spectroscopy data improves detection of prostate cancer in tissue specimens | 2021 |
| Discrimination of breast cancer from benign tumours using Raman spectroscopy | 2019 |
| Employing Raman Spectroscopy and Machine Learning for the Identification of Breast Cancer | 2024 |
| Granulocyte colony-stimulating factor promotes an aggressive phenotype of colon and breast cancer cells with biochemical changes investigated by single-cell Raman microspectroscopy and machine learning analysis | 2021 |
| Grouped-sampling technique to deal with unbalance in Raman spectral data modeling | 2022 |
| Identification of Molecular Basis for Objective Discrimination of Breast Cancer Cells (MCF-7) from Normal Human Mammary Epithelial Cells by Raman Microspectroscopy and Multivariate Curve Resolution Analysis | 2021 |
| In situ Raman spectroscopy and machine learning unveil biomolecular alterations in invasive breast cancer | 2023 |
| Label-free diagnosis for colorectal cancer through coffee ring-assisted surface-enhanced Raman spectroscopy on blood serum | 2020 |
| Label-Free Identification of Exosomes using Raman Spectroscopy and Machine Learning | 2023 |
| Label-Free Plasmon-Enhanced Spectroscopic HER2 Detection for Dynamic Therapeutic Surveillance of Breast Cancer | 2022 |
| Label-Free Vibrational and Quantitative Phase Microscopy Reveals Remarkable Pathogen-Induced Morphomolecular Divergence in Tumor-Derived Cells | 2022 |
| Low coherence quantitative phase microscopy with machine learning model and Raman spectroscopy for the study of breast cancer cells and their classification | 2019 |
| Machine learning assisted dual-modal SERS detection for circulating tumor cells | 2025 |
| Machine Learning Assisted Real-Time Label-Free SERS Diagnoses of Malignant Pleural Effusion due to Lung Cancer | 2022 |
| Machine learning characterization of cancer patients-derived extracellular vesicles using vibrational spectroscopies: results from a pilot study. | 2022 |
| Raman spectroscopy combined with multivariate statistical algorithms for the simultaneous screening of cervical and breast cancers | 2024 |
| Machine Learning-Based Label-Free SERS Profiling of Exosomes for Accurate Fuzzy Diagnosis of Cancer and Dynamic Monitoring of Drug Therapeutic Processes | 2023 |
| Macroscopic inelastic scattering imaging using a hyperspectral line-scanning system identifies invasive breast cancer in lumpectomy and mastectomy specimens | 2024 |
| Method for accurate registration of tissue autofluorescence imaging data with corresponding histology: a means for enhanced tumor margin assessment | 2018 |
| Minimal information for studies of extracellular vesicles (MISEV2023): From basic to advanced approaches | 2024 |
| Multiplexed Surface Protein Detection and Cancer Classification Using Gap-Enhanced Magnetic-Plasmonic Core-Shell Raman Nanotags and Machine Learning Algorithm | 2024 |
| Non-invasive screening and subtyping for breast cancer by serum SERS combined with LGB-DNN algorithms | 2024 |
| Optical parameters of healthy and tumor breast tissues in mice | 2024 |
| Polarized Micro-Raman Spectroscopy and 2D Convolutional Neural Network Applied to Structural Analysis and Discrimination of Breast Cancer | 2023 |
| Polysulfide Serves as a Hallmark of Desmoplastic Reaction to Differentially Diagnose Ductal Carcinoma In Situ and Invasive Breast Cancer by SERS Imaging | 2023 |
| Profiling Breast Tumor Heterogeneity and Identifying Breast Cancer Subtypes Through Tumor-Associated Immune Cell Signatures and Immuno Nano Sensors | 2024 |
| Quantum cytosensor for early detection of cancer | 2020 |
| Radiation treatment response and hypoxia biomarkers revealed by machine learning assisted Raman spectroscopy in tumour cells and xenograft tissues | 2022 |
| Raman microspectroscopy and machine learning for use in identifying radiation-induced lung toxicity | 2022 |
| Raman optical identification of renal cell carcinoma via machine learning | 2021 |
| Raman spectral pattern recognition of breast cancer: A machine learning strategy based on feature fusion and adaptive hyperparameter optimization | 2023 |
| Raman Spectroscopy and AI Applications in Cancer Grading: An Overview | 2024 |
| Raman spectroscopy and artificial intelligence to predict the Bayesian probability of breast cancer | 2021 |
| Raman spectroscopy and convolutional neural networks for monitoring biochemical radiation response in breast tumour xenografts | 2023 |
| Raman spectroscopy and group and basis-restricted non negative matrix factorisation identifies radiation induced metabolic changes in human cancer cells | 2021 |
| Raman spectroscopy and machine learning for the classification of breast cancers | 2022 |
| Raman spectroscopy combined with convolutional neural network for the sub-types classification of breast cancer and critical feature visualization | 2024 |
| Raman spectroscopy reveals phenotype switches in breast cancer metastasis | 2022 |
| Raman Spectroscopy: A Personalized Decision-Making Tool on Clinicians' Hands for In Situ Cancer Diagnosis and Surgery Guidance | 2022 |
| Rapid Diagnosis of Ductal Carcinoma In Situ and Breast Cancer Based on Raman Spectroscopy of Serum Combined with Convolutional Neural Network | 2023 |
| Rapid identification of papillary thyroid carcinoma and papillary microcarcinoma based on serum Raman spectroscopy combined with machine learning models | 2022 |
| Rapid multi-task diagnosis of oral cancer leveraging fiber-optic Raman spectroscopy and deep learning algorithms | 2023 |
| Robot-assisted biopsy sampling for online Raman spectroscopy cancer confirmation in the operating room | 2024 |
| Robust Diagnosis of Breast Cancer Based on Silver Nanoparticles by Surface-Enhanced Raman Spectroscopy and Machine Learning | 2024 |
| Role of artificial intelligence and vibrational spectroscopy in cancer diagnostics | 2020 |
| Scalable nanolaminated SERS multiwell cell culture assay | 2020 |
| SERS liquid biopsy in breast cancer. What can we learn from SERS on serum and urine? | 2022 |
| Serum analysis based on SERS combined with 2D convolutional neural network and Gramian angular field for breast cancer screening | 2024 |
| Serum Raman spectroscopy combined with multiple classification models for rapid diagnosis of breast cancer | 2022 |
| Single-Cell Analysis and Classification according to Multiplexed Proteins via Microdroplet-Based Self-Driven Magnetic Surface-Enhanced Raman Spectroscopy Platforms Assisted with Machine Learning Algorithms | 2023 |
| Space curvature-inspired nanoplasmonic sensor for breast cancer extracellular vesicle fingerprinting and machine learning classification | 2021 |
| Toward noncontact macroscopic imaging of multiple cancers using multi-spectral inelastic scattering detection | 2024 |

1. References:
   Nguyen, T. M., Jeong, S., Kang, S. K., Han, S. W., Nguyen, T. M. T., Lee, S., Jung, Y. J., Kim, Y. H., Park, S., Bak, G. H., Ko, Y. C., Choi, E. J., Kim, H. Y., & Oh, J. W. (2024). 3D Superclusters with Hybrid Bioinks for Early Detection in Breast Cancer. *ACS sensors*, *9*(2), 699–707. <https://doi.org/10.1021/acssensors.3c01938>
2. Jiang, J., Li, L., Yin, G., Luo, H., & Li, J. (2024). A Molecular Typing Method for Invasive Breast Cancer by Serum Raman Spectroscopy. *Clinical breast cancer*, *24*(4), 376–383. <https://doi.org/10.1016/j.clbc.2024.02.008>
3. Qi, Y., Yang, L., Liu, B., Liu, L., Liu, Y., Zheng, Q., Liu, D., & Luo, J. (2021). Accurate diagnosis of lung tissues for 2D Raman spectrogram by deep learning based on short-time Fourier transform. *Analytica chimica acta*, *1179*, 338821. <https://doi.org/10.1016/j.aca.2021.338821>
4. Sezer, G., Sahin, F., Onses, M. S., & Cumaoglu, A. (2024). Activation of epidermal growth factor receptors in triple-negative breast cancer cells by morphine; analysis through Raman spectroscopy and machine learning. *Talanta*, *272*, 125827. <https://doi.org/10.1016/j.talanta.2024.125827>
5. Talari, A. C. S., Rehman, S., & Rehman, I. U. (2019). Advancing cancer diagnostics with artificial intelligence and spectroscopy: identifying chemical changes associated with breast cancer. *Expert review of molecular diagnostics*, *19*(10), 929–940. <https://doi.org/10.1080/14737159.2019.1659727>
6. Lin, R., Peng, B., Li, L., He, X., Yan, H., Tian, C., Luo, H., & Yin, G. (2023). Application of serum Raman spectroscopy combined with classification model for rapid breast cancer screening. *Frontiers in oncology*, *13*, 1258436. <https://doi.org/10.3389/fonc.2023.1258436>
7. Cheng, Z., Li, H., Chen, C., Lv, X., Zuo, E., Xie, X., Li, Z., Liu, P., Li, H., & Chen, C. (2023). Application of serum SERS technology based on thermally annealed silver nanoparticle composite substrate in breast cancer. *Photodiagnosis and photodynamic therapy*, *41*, 103284. <https://doi.org/10.1016/j.pdpdt.2023.103284>
8. Haldavnekar, R., Venkatakrishnan, K., & Tan, B. (2022). Cancer Stem Cell Derived Extracellular Vesicles with Self-Functionalized 3D Nanosensor for Real-Time Cancer Diagnosis: Eliminating the Roadblocks in Liquid Biopsy. *ACS nano*, *16*(8), 12226–12243. <https://doi.org/10.1021/acsnano.2c02971>
9. Liu, C., Xiu, C., Zou, Y., Wu, W., Huang, Y., Wan, L., Xu, S., Han, B., & Zhang, H. (2025). Cervical cancer diagnosis model using spontaneous Raman and Coherent anti-Stokes Raman spectroscopy with artificial intelligence. *Spectrochimica acta. Part A, Molecular and biomolecular spectroscopy*, *327*, 125353. <https://doi.org/10.1016/j.saa.2024.125353>
10. Ma, D., Shang, L., Tang, J., Bao, Y., Fu, J., & Yin, J. (2021). Classifying breast cancer tissue by Raman spectroscopy with one-dimensional convolutional neural network. *Spectrochimica acta. Part A, Molecular and biomolecular spectroscopy*, *256*, 119732. <https://doi.org/10.1016/j.saa.2021.119732>
11. Lee, W., Lenferink, A. T., Otto, C., & Offerhaus, H. L. (2020). Classifying Raman spectra of extracellular vesicles based on convolutional neural networks for prostate cancer detection. *Journal of raman spectroscopy*, *51*(2), 293-300. [**https://doi.org/10.1002/jrs.5770**](https://doi.org/10.1002/jrs.5770)
12. Bratchenko, I. A., & Bratchenko, L. A. (2023). Comment on "Serum Raman spectroscopy combined with multiple classification models for rapid diagnosis of breast cancer". *Photodiagnosis and photodynamic therapy*, *41*, 103215. <https://doi.org/10.1016/j.pdpdt.2022.103215>
13. Kazemzadeh, M., Hisey, C. L., Zargar-Shoshtari, K., Xu, W., & Broderick, N. G. (2022). Deep convolutional neural networks as a unified solution for Raman spectroscopy-based classification in biomedical applications. *Optics Communications*, *510*, 127977. <https://doi.org/10.1016/j.optcom.2022.127977>
14. Kim, J., Son, H. Y., Lee, S., Rho, H. W., Kim, R., Jeong, H., Park, C., Mun, B., Moon, Y., Jeong, E., Lim, E. K., & Haam, S. (2024). Deep learning-assisted monitoring of trastuzumab efficacy in HER2-Overexpressing breast cancer via SERS immunoassays of tumor-derived urinary exosomal biomarkers. *Biosensors & bioelectronics*, *258*, 116347. <https://doi.org/10.1016/j.bios.2024.116347>
15. Tipatet, K. S., Davison-Gates, L., Tewes, T. J., Fiagbedzi, E. K., Elfick, A., Neu, B., & Downes, A. (2021). Detection of acquired radioresistance in breast cancer cell lines using Raman spectroscopy and machine learning. *The Analyst*, *146*(11), 3709–3716. <https://doi.org/10.1039/d1an00387a>
16. Mathew, A. P., Cutshaw, G., Appel, O., Funk, M., Synan, L., Waite, J., Ghazvini, S., Wen, X., Sarkar, S., Santillan, M., Santillan, D., & Bardhan, R. (2024). Diagnosis of pregnancy disorder in the first-trimester patient plasma with Raman spectroscopy and protein analysis. *Bioengineering & translational medicine*, *9*(6), e10691. <https://doi.org/10.1002/btm2.10691>
17. Plante, A., Dallaire, F., Grosset, A. A., Nguyen, T., Birlea, M., Wong, J., Daoust, F., Roy, N., Kougioumoutzakis, A., Azzi, F., Aubertin, K., Kadoury, S., Latour, M., Albadine, R., Prendeville, S., Boutros, P., Fraser, M., Bristow, R. G., van der Kwast, T., Orain, M., … Leblond, F. (2021). Dimensional reduction based on peak fitting of Raman micro spectroscopy data improves detection of prostate cancer in tissue specimens. *Journal of biomedical optics*, *26*(11), 116501. <https://doi.org/10.1117/1.JBO.26.11.116501>
18. Lyng, F. M., Traynor, D., Nguyen, T. N. Q., Meade, A. D., Rakib, F., Al-Saady, R., Goormaghtigh, E., Al-Saad, K., & Ali, M. H. (2019). Discrimination of breast cancer from benign tumours using Raman spectroscopy. *PloS one*, *14*(2), e0212376. <https://doi.org/10.1371/journal.pone.0212376>
19. Zhang, Y., Li, Z., Li, Z., Wang, H., Regmi, D., Zhang, J., Feng, J., Yao, S., & Xu, J. (2024). Employing Raman Spectroscopy and Machine Learning for the Identification of Breast Cancer. *Biological procedures online*, *26*(1), 28. <https://doi.org/10.1186/s12575-024-00255-0>
20. Zhang, W., Karagiannidis, I., Van Vliet, E. S., Yao, R., Beswick, E. J., & Zhou, A. (2021). Granulocyte colony-stimulating factor promotes an aggressive phenotype of colon and breast cancer cells with biochemical changes investigated by single-cell Raman microspectroscopy and machine learning analysis. *The Analyst*, *146*(20), 6124–6131. <https://doi.org/10.1039/d1an00938a>
21. Song, H., Leng, H., Hou, Z., Gao, R., Chen, C., Meng, C., Sun, J., Li, C., & Ma, B. (2022). Grouped-sampling technique to deal with unbalance in Raman spectral data modeling. *Photodiagnosis and photodynamic therapy*, *40*, 103059. <https://doi.org/10.1016/j.pdpdt.2022.103059>
22. Iwasaki, K., Araki, A., Krishna, C. M., Maruyama, R., Yamamoto, T., & Noothalapati, H. (2021). Identification of Molecular Basis for Objective Discrimination of Breast Cancer Cells (MCF-7) from Normal Human Mammary Epithelial Cells by Raman Microspectroscopy and Multivariate Curve Resolution Analysis. *International journal of molecular sciences*, *22*(2), 800. <https://doi.org/10.3390/ijms22020800>
23. David, S., Tran, T., Dallaire, F., Sheehy, G., Azzi, F., Trudel, D., Tremblay, F., Omeroglu, A., Leblond, F., & Meterissian, S. (2023). *In situ* Raman spectroscopy and machine learning unveil biomolecular alterations in invasive breast cancer. *Journal of biomedical optics*, *28*(3), 036009. <https://doi.org/10.1117/1.JBO.28.3.036009>
24. Hong, Y., Li, Y., Huang, L., He, W., Wang, S., Wang, C., Zhou, G., Chen, Y., Zhou, X., Huang, Y., Huang, W., Gong, T., & Zhou, Z. (2020). Label-free diagnosis for colorectal cancer through coffee ring-assisted surface-enhanced Raman spectroscopy on blood serum. *Journal of biophotonics*, *13*(4), e201960176. <https://doi.org/10.1002/jbio.201960176>
25. Parlatan, U., Ozen, M. O., Kecoglu, I., Koyuncu, B., Torun, H., Khalafkhany, D., Loc, I., Ogut, M. G., Inci, F., Akin, D., Solaroglu, I., Ozoren, N., Unlu, M. B., & Demirci, U. (2023). Label-Free Identification of Exosomes using Raman Spectroscopy and Machine Learning. *Small (Weinheim an der Bergstrasse, Germany)*, *19*(9), e2205519. <https://doi.org/10.1002/smll.202205519>
26. Xie, Y., Wen, Y., Su, X., Zheng, C., & Li, M. (2022). Label-Free Plasmon-Enhanced Spectroscopic HER2 Detection for Dynamic Therapeutic Surveillance of Breast Cancer. *Analytical chemistry*, *94*(37), 12762–12771. <https://doi.org/10.1021/acs.analchem.2c02419>
27. Liu, Z., Parida, S., Wu, S., Sears, C. L., Sharma, D., & Barman, I. (2022). Label-Free Vibrational and Quantitative Phase Microscopy Reveals Remarkable Pathogen-Induced Morphomolecular Divergence in Tumor-Derived Cells. *ACS sensors*, *7*(5), 1495–1505. <https://doi.org/10.1021/acssensors.2c00232>
28. Dubey, V., Ahmad, A., Butola, A., Qaiser, D., Srivastava, A., & Mehta, D. S. (2019). Low coherence quantitative phase microscopy with machine learning model and Raman spectroscopy for the study of breast cancer cells and their classification. *Applied optics*, *58*(5), A112–A119. <https://doi.org/10.1364/AO.58.00A112>
29. Zhang, C., Xu, L., Miao, X., Zhang, D., Xie, Y., Hu, Y., Zhang, Z., Wang, X., Wu, X., Liu, Z., Zang, W., He, C., Li, Z., Ren, W., Chen, T., Xu, C., Zhang, Y., Wu, A., & Lin, J. (2025). Machine learning assisted dual-modal SERS detection for circulating tumor cells. *Biosensors & bioelectronics*, *268*, 116897. <https://doi.org/10.1016/j.bios.2024.116897>
30. Perumal, J., Lee, P., Dev, K., Lim, H. Q., Dinish, U. S., & Olivo, M. (2022). Machine Learning Assisted Real-Time Label-Free SERS Diagnoses of Malignant Pleural Effusion due to Lung Cancer. *Biosensors*, *12*(11), 940. <https://doi.org/10.3390/bios12110940>
31. Uthamacumaran, A., Elouatik, S., Abdouh, M., Berteau-Rainville, M., Gao, Z. H., & Arena, G. (2022). Machine learning characterization of cancer patients-derived extracellular vesicles using vibrational spectroscopies: results from a pilot study. *Applied Intelligence*, *52*(11), 12737-12753. <https://doi.org/10.1007/s10489-022-03203-1>
32. Klamminger, G. G., Mombaerts, L., Kemp, F., Jelke, F., Klein, K., Slimani, R., Mirizzi, G., Husch, A., Hertel, F., Mittelbronn, M., & Kleine Borgmann, F. B. (2024). Machine Learning-Assisted Classification of Paraffin-Embedded Brain Tumors with Raman Spectroscopy. *Brain sciences*, *14*(4), 301. <https://doi.org/10.3390/brainsci14040301>
33. Diao, X., Li, X., Hou, S., Li, H., Qi, G., & Jin, Y. (2023). Machine Learning-Based Label-Free SERS Profiling of Exosomes for Accurate Fuzzy Diagnosis of Cancer and Dynamic Monitoring of Drug Therapeutic Processes. *Analytical chemistry*, *95*(19), 7552–7559. <https://doi.org/10.1021/acs.analchem.3c00026>
34. David, S., Tavera, H., Trang, T., Dallaire, F., Daoust, F., Tremblay, F., Richer, L., Meterissian, S., & Leblond, F. (2024). Macroscopic inelastic scattering imaging using a hyperspectral line-scanning system identifies invasive breast cancer in lumpectomy and mastectomy specimens. *Journal of biomedical optics*, *29*(6), 065004. <https://doi.org/10.1117/1.JBO.29.6.065004>
35. Unger, J., Sun, T., Chen, Y. L., Phipps, J. E., Bold, R. J., Darrow, M. A., Ma, K. L., & Marcu, L. (2018). Method for accurate registration of tissue autofluorescence imaging data with corresponding histology: a means for enhanced tumor margin assessment. *Journal of biomedical optics*, *23*(1), 1–11. <https://doi.org/10.1117/1.JBO.23.1.015001>
36. Welsh, J. A., Goberdhan, D. C. I., O'Driscoll, L., Buzas, E. I., Blenkiron, C., Bussolati, B., Cai, H., Di Vizio, D., Driedonks, T. A. P., Erdbrügger, U., Falcon-Perez, J. M., Fu, Q. L., Hill, A. F., Lenassi, M., Lim, S. K., Mahoney, M. G., Mohanty, S., Möller, A., Nieuwland, R., Ochiya, T., … Witwer, K. W. (2024). Minimal information for studies of extracellular vesicles (MISEV2023): From basic to advanced approaches. *Journal of extracellular vesicles*, *13*(2), e12404. <https://doi.org/10.1002/jev2.12404>
37. Rodriguez-Nieves, A. L., Taylor, M. L., Wilson, R., Eldridge, B. K., Nawalage, S., Annamer, A., Miller, H. G., Alle, M. R., Gomrok, S., Zhang, D., Wang, Y., & Huang, X. (2024). Multiplexed Surface Protein Detection and Cancer Classification Using Gap-Enhanced Magnetic-Plasmonic Core-Shell Raman Nanotags and Machine Learning Algorithm. *ACS applied materials & interfaces*, *16*(2), 2041–2057. <https://doi.org/10.1021/acsami.3c13921>
38. Zhang, Q., Lin, Y., Lin, D., Lin, X., Liu, M., Tao, H., Wu, J., Wang, T., Wang, C., & Feng, S. (2024). Non-invasive screening and subtyping for breast cancer by serum SERS combined with LGB-DNN algorithms. *Talanta*, *275*, 126136. <https://doi.org/10.1016/j.talanta.2024.126136>
39. Genina, E. A., Lazareva, E. N., Surkov, Y. I., Serebryakova, I. A., & Shushunova, N. A. (2024). Optical parameters of healthy and tumor breast tissues in mice. *Journal of biophotonics*, *17*(8), e202400123. <https://doi.org/10.1002/jbio.202400123>
40. Shang, L., Tang, J., Wu, J., Shang, H., Huang, X., Bao, Y., Xu, Z., Wang, H., & Yin, J. (2022). Polarized Micro-Raman Spectroscopy and 2D Convolutional Neural Network Applied to Structural Analysis and Discrimination of Breast Cancer. *Biosensors*, *13*(1), 65. <https://doi.org/10.3390/bios13010065>
41. Kubo, A., Masugi, Y., Hase, T., Nagashima, K., Kawai, Y., Takizawa, M., Hishiki, T., Shiota, M., Wakui, M., Kitagawa, Y., Kabe, Y., Sakamoto, M., Yachie, A., Hayashida, T., & Suematsu, M. (2023). Polysulfide Serves as a Hallmark of Desmoplastic Reaction to Differentially Diagnose Ductal Carcinoma In Situ and Invasive Breast Cancer by SERS Imaging. *Antioxidants (Basel, Switzerland)*, *12*(2), 240. <https://doi.org/10.3390/antiox12020240>
42. Ishwar, D., Premachandran, S., Das, S., Venkatakrishnan, K., & Tan, B. (2024). Profiling Breast Tumor Heterogeneity and Identifying Breast Cancer Subtypes Through Tumor-Associated Immune Cell Signatures and Immuno Nano Sensors. *Small (Weinheim an der Bergstrasse, Germany)*, *20*(52), e2406475. <https://doi.org/10.1002/smll.202406475>
43. Ganesh, S., Venkatakrishnan, K., & Tan, B. (2020). Quantum cytosensor for early detection of cancer. *Medical Devices & Sensors*, *3*(1), e10058. [**https://doi.org/10.1002/mds3.10058**](https://doi.org/10.1002/mds3.10058)
44. Deng, X., Milligan, K., Brolo, A., Lum, J. J., Andrews, J. L., & Jirasek, A. (2022). Radiation treatment response and hypoxia biomarkers revealed by machine learning assisted Raman spectroscopy in tumour cells and xenograft tissues. *The Analyst*, *147*(22), 5091–5104. <https://doi.org/10.1039/d2an01222g>
45. Ali-Adeeb, R. N., Shreeves, P., Deng, X., Milligan, K., Brolo, A. G., Lum, J. J., Haston, C., Andrews, J. L., & Jirasek, A. (2022). Raman microspectroscopy and machine learning for use in identifying radiation-induced lung toxicity. *PloS one*, *17*(12), e0279739. <https://doi.org/10.1371/journal.pone.0279739>
46. He, C., Wu, X., Zhou, J., Chen, Y., & Ye, J. (2021). Raman optical identification of renal cell carcinoma via machine learning. *Spectrochimica acta. Part A, Molecular and biomolecular spectroscopy*, *252*, 119520. <https://doi.org/10.1016/j.saa.2021.119520>
47. Li, Q., Zhang, Z., & Ma, Z. (2023). Raman spectral pattern recognition of breast cancer: A machine learning strategy based on feature fusion and adaptive hyperparameter optimization. *Heliyon*, *9*(7), e18148. <https://doi.org/10.1016/j.heliyon.2023.e18148>
48. Conforti, P. M., Lazzini, G., Russo, P., & D’Acunto, M. (2024). Raman spectroscopy and AI applications in cancer grading. An overview. *IEEE Access*. https://doi: 10.1109/ACCESS.2024.3388841
49. Kothari, R., Jones, V., Mena, D., Bermúdez Reyes, V., Shon, Y., Smith, J. P., Schmolze, D., Cha, P. D., Lai, L., Fong, Y., & Storrie-Lombardi, M. C. (2021). Raman spectroscopy and artificial intelligence to predict the Bayesian probability of breast cancer. *Scientific reports*, *11*(1), 6482. <https://doi.org/10.1038/s41598-021-85758-6>
50. Fuentes, A. M., Narayan, A., Milligan, K., Lum, J. J., Brolo, A. G., Andrews, J. L., & Jirasek, A. (2023). Raman spectroscopy and convolutional neural networks for monitoring biochemical radiation response in breast tumour xenografts. *Scientific reports*, *13*(1), 1530. <https://doi.org/10.1038/s41598-023-28479-2>
51. Milligan, K., Deng, X., Shreeves, P., Ali-Adeeb, R., Matthews, Q., Brolo, A., Lum, J. J., Andrews, J. L., & Jirasek, A. (2021). Raman spectroscopy and group and basis-restricted non negative matrix factorisation identifies radiation induced metabolic changes in human cancer cells. *Scientific reports*, *11*(1), 3853. <https://doi.org/10.1038/s41598-021-83343-5>
52. Zhang, L., Li, C., Peng, D., Yi, X., He, S., Liu, F., Zheng, X., Huang, W. E., Zhao, L., & Huang, X. (2022). Raman spectroscopy and machine learning for the classification of breast cancers. *Spectrochimica acta. Part A, Molecular and biomolecular spectroscopy*, *264*, 120300. <https://doi.org/10.1016/j.saa.2021.120300>
53. Li, J., Wang, X., Min, S., Xia, J., & Li, J. (2024). Raman spectroscopy combined with convolutional neural network for the sub-types classification of breast cancer and critical feature visualization. *Computer methods and programs in biomedicine*, *255*, 108361. <https://doi.org/10.1016/j.cmpb.2024.108361>
54. Paidi, S. K., Troncoso, J. R., Harper, M. G., Liu, Z., Nguyen, K. G., Ravindranathan, S., Rebello, L., Lee, D. E., Ivers, J. D., Zaharoff, D. A., Rajaram, N., & Barman, I. (2022). Raman spectroscopy reveals phenotype switches in breast cancer metastasis. *Theranostics*, *12*(12), 5351–5363. <https://doi.org/10.7150/thno.74002>
55. Kouri, M. A., Spyratou, E., Karnachoriti, M., Kalatzis, D., Danias, N., Arkadopoulos, N., Seimenis, I., Raptis, Y. S., Kontos, A. G., & Efstathopoulos, E. P. (2022). Raman Spectroscopy: A Personalized Decision-Making Tool on Clinicians' Hands for In Situ Cancer Diagnosis and Surgery Guidance. *Cancers*, *14*(5), 1144. <https://doi.org/10.3390/cancers14051144>
56. Wang, X., Xie, F., Yang, Y., Zhao, J., Wu, G., & Wang, S. (2023). Rapid Diagnosis of Ductal Carcinoma In Situ and Breast Cancer Based on Raman Spectroscopy of Serum Combined with Convolutional Neural Network. *Bioengineering (Basel, Switzerland)*, *10*(1), 65. <https://doi.org/10.3390/bioengineering10010065>
57. Song, H., Dong, C., Zhang, X., Wu, W., Chen, C., Ma, B., Chen, F., Chen, C., & Lv, X. (2022). Rapid identification of papillary thyroid carcinoma and papillary microcarcinoma based on serum Raman spectroscopy combined with machine learning models. *Photodiagnosis and photodynamic therapy*, *37*, 102647. <https://doi.org/10.1016/j.pdpdt.2021.102647>
58. Li, X., Li, L., Sun, Q., Chen, B., Zhao, C., Dong, Y., Zhu, Z., Zhao, R., Ma, X., Yu, M., & Zhang, T. (2023). Rapid multi-task diagnosis of oral cancer leveraging fiber-optic Raman spectroscopy and deep learning algorithms. *Frontiers in oncology*, *13*, 1272305. <https://doi.org/10.3389/fonc.2023.1272305>
59. Grajales, D., Le, W. T., Tran, T., David, S., Dallaire, F., Ember, K., Leblond, F., Ménard, C., & Kadoury, S. (2024). Robot-assisted biopsy sampling for online Raman spectroscopy cancer confirmation in the operating room. *International journal of computer assisted radiology and surgery*, *19*(6), 1103–1111. <https://doi.org/10.1007/s11548-024-03100-7>
60. Wang, M., Zhang, K., Yue, L., Liu, X., Lai, Y., & Zhang, H. (2024). Robust Diagnosis of Breast Cancer Based on Silver Nanoparticles by Surface-Enhanced Raman Spectroscopy and Machine Learning. *ACS Applied Nano Materials*, *7*(11), 13672-13680. <https://doi.org/10.1021/acsanm.4c02191>
61. Rehman, I. U., Khan, R. S., & Rehman, S. (2020). Role of artificial intelligence and vibrational spectroscopy in cancer diagnostics. *Expert review of molecular diagnostics*, *20*(8), 749–755. <https://doi.org/10.1080/14737159.2020.1784008>
62. Ren, X., Nam, W., Ghassemi, P., Strobl, J. S., Kim, I., Zhou, W., & Agah, M. (2020). Scalable nanolaminated SERS multiwell cell culture assay. *Microsystems & nanoengineering*, *6*, 47. <https://doi.org/10.1038/s41378-020-0145-3>
63. Iancu, S. D., Cozan, R. G., Stefancu, A., David, M., Moisoiu, T., Moroz-Dubenco, C., Bajcsi, A., Chira, C., Andreica, A., Leopold, L. F., Eniu, D., Staicu, A., Goidescu, I., Socaciu, C., Eniu, D. T., Diosan, L., & Leopold, N. (2022). SERS liquid biopsy in breast cancer. What can we learn from SERS on serum and urine?. *Spectrochimica acta. Part A, Molecular and biomolecular spectroscopy*, *273*, 120992. <https://doi.org/10.1016/j.saa.2022.120992>
64. Cheng, N., Gao, Y., Ju, S., Kong, X., Lyu, J., Hou, L., Jin, L., & Shen, B. (2024). Serum analysis based on SERS combined with 2D convolutional neural network and Gramian angular field for breast cancer screening. *Spectrochimica acta. Part A, Molecular and biomolecular spectroscopy*, *312*, 124054. <https://doi.org/10.1016/j.saa.2024.124054>
65. Li, H., Wang, S., Zeng, Q., Chen, C., Lv, X., Ma, M., Su, H., Ma, B., Chen, C., & Fang, J. (2022). Serum Raman spectroscopy combined with multiple classification models for rapid diagnosis of breast cancer. *Photodiagnosis and photodynamic therapy*, *40*, 103115. <https://doi.org/10.1016/j.pdpdt.2022.103115>
66. Wang, J., Cong, L., Shi, W., Xu, W., & Xu, S. (2023). Single-Cell Analysis and Classification according to Multiplexed Proteins via Microdroplet-Based Self-Driven Magnetic Surface-Enhanced Raman Spectroscopy Platforms Assisted with Machine Learning Algorithms. *Analytical chemistry*, *95*(29), 11019–11027. <https://doi.org/10.1021/acs.analchem.3c01273>
67. Kazemzadeh, M., Hisey, C. L., Artuyants, A., Blenkiron, C., Chamley, L. W., Zargar-Shoshtari, K., Xu, W., & Broderick, N. G. R. (2021). Space curvature-inspired nanoplasmonic sensor for breast cancer extracellular vesicle fingerprinting and machine learning classification. *Biomedical optics express*, *12*(7), 3965–3981. <https://doi.org/10.1364/BOE.428302>
68. David, S., Ksantini, N., Dallaire, F., Ember, K., Daoust, F., Sheehy, G., Hadjipanayis, C. G., Petrecca, K., Wilson, B. C., & Leblond, F. (2024). Toward noncontact macroscopic imaging of multiple cancers using multi-spectral inelastic scattering detection. *Journal of biophotonics*, *17*(9), e202400087. <https://doi.org/10.1002/jbio.202400087>
